# Expression of Putative Defense Responses in Cannabis Primed by *Pseudomonas* and/or *Bacillus* Strains and Infected by *Botrytis cinerea*

**DOI:** 10.3389/fpls.2020.572112

**Published:** 2020-11-25

**Authors:** Carole Balthazar, Gabrielle Cantin, Amy Novinscak, David L. Joly, Martin Filion

**Affiliations:** ^1^Department of Biology, Université de Moncton, Moncton, NB, Canada; ^2^Institute of Health Sciences, Collège La Cité, Ottawa, ON, Canada; ^3^Agriculture and Agri-Food Canada, Saint-Jean-sur-Richelieu Research and Development Centre, Saint-Jean-sur-Richelieu, QC, Canada

**Keywords:** *Pseudomonas*, *Bacillus*, plant growth promoting rhizobacteria, systemic acquired resistance, induced systemic resistance, *Cannabis sativa*, gray mold, *Botrytis cinerea*

## Abstract

Cannabis (*Cannabis sativa* L.) offers many industrial, agricultural, and medicinal applications, but is commonly threatened by the gray mold disease caused by the fungus *Botrytis cinerea*. With few effective control measures currently available, the use of beneficial rhizobacteria represents a promising biocontrol avenue for cannabis. To counter disease development, plants rely on a complex network of inducible defense pathways, allowing them to respond locally and systemically to pathogens attacks. In this study, we present the first attempt to control gray mold in cannabis using beneficial rhizobacteria, and the first investigation of cannabis defense responses at the molecular level. Four promising *Pseudomonas* (LBUM223 and WCS417r) and *Bacillus* strains (LBUM279 and LBUM979) were applied as single or combined root treatments to cannabis seedlings, which were subsequently infected by *B. cinerea.* Symptoms were recorded and the expression of eight putative defense genes was monitored in leaves by reverse transcription quantitative polymerase chain reaction. The rhizobacteria did not significantly control gray mold and all infected leaves were necrotic after a week, regardless of the treatment. Similarly, no systemic activation of putative cannabis defense genes was reported, neither triggered by the pathogen nor by the rhizobacteria. However, this work identified five putative defense genes (*ERF1*, *HEL*, *PAL*, *PR1*, and *PR2*) that were strongly and sustainably induced locally at *B. cinerea*’s infection sites, as well as two stably expressed reference genes (*TIP41* and *APT1*) in cannabis. These markers will be useful in future researches exploring cannabis defense pathways.

## Introduction

The cannabis plant, *Cannabis sativa* L., is an annual herbaceous plant belonging to the Cannabaceae family. Probably domesticated in Central Asia thousands of years ago, its great ecological range and its interaction with humans have allowed it to spread throughout the world as cultivated and wild populations (Lynch et al., 2016). The female inflorescences of cannabis are covered with glandular trichomes containing numerous aromatic secondary metabolites, including terpenes and phytocannabinoids. Among these, tetrahydrocannabinol (THC) is the main psychoactive compound and its dry mass content is used to discriminate between marijuana cultivars (cultivated for psychoactive/medicinal substances) and hemp cultivars (cultivated for fibers, seeds and oil) with an arbitrary threshold of 0.3% (Small and Cronquist, 1976). As legislations allowing marijuana use evolve around the world, hemp cultivation is also drawing more attention to produce non-psychoactive medicinal substances (Conant et al., 2017). Despite its multi-billion dollar worth to one of the fastest growing industries in North America, this plant remains poorly understood compared to other economically important crops, mainly because of its legal constraints (Vergara et al., 2016).

Close to ninety different fungal species can cause diseases on cannabis. Among them, one of the most important is *Botrytis cinerea*, causing gray mold. This air-borne necrotrophic fungus can infect seeds, leaves, inflorescences and stalks, forming spreading lesions covered by a gray mat of thousands of conidia and eventually leading to a rapid decay of the plant and polycyclic epidemics (McPartland et al., 2000). *B. cinerea* can reduce outdoor hemp yield by 32% during rainy years (Van der Werf et al., 1995) and also causes significant quality issues and post-harvest losses in indoor facilities since contaminated marijuana buds are unfit for consumption (Punja et al., 2019). Moreover, aerial conidia may expose cannabis workers to occupational health hazards such as allergic sensitization and hypersensitivity pneumonitis, especially in outdoor production farms where *B. cinerea* was reported as the most prevalent fungus accounting for 34% of all fungi detected in air samples (Green et al., 2018). *B. cinerea* also attacks over two hundred hosts worldwide, including major vegetable crops, legumes, berries, and ornamental plants, and is the most studied necrotrophic pathogen with a broad host range. Its infection strategy includes the secretion of lytic enzymes to breach the plant surface, followed by the synthesis of phytotoxic metabolites to trigger an oxidative burst and the induction of host programmed cell death. Successfully killing the plant cells allows the subsequent maceration of dead tissues to extract nutrients for fungal growth (van Kan, 2006).

With global control measures costs exceeding one billion euros per year in 2012, *B. cinerea* has earned the title of the second most economically important pathogenic fungus worldwide behind *Magnaporthe oryzae* (Dean et al., 2012). In most crops, the primary control method against gray mold remains the application of synthetic fungicides with various modes of action targeting respiration, cytoskeleton assembly, osmoregulation, sterol and amino-acid biosynthesis. However, fungicide-mediated selection pressure has led to the problematic emergence of fungal isolates overcoming susceptibility thanks to their metabolic detoxification capabilities, upregulated efflux membrane transporters or modified target sites (Fillinger and Walker, 2016). Besides, use of fungicides on cannabis crops is stringently regulated by law in most countries due to health and environmental effects (Seltenrich, 2019). Another control option relies on cultivar breeding for resistance against *B. cinerea*, for example by abolishing plant programmed cell death responses. Unfortunately, this strategy may in turn compromise the plant’s resistance against biotrophic pathogens (Williamson et al., 2007), like *Golovinomyces* spp. causing powdery mildew, which is another prevalent fungal disease in cannabis (Pépin et al., 2018). Finally, gray mold incidence can be mitigated by integrating cultural practices adapted to specific indoor/outdoor cropping systems. In controlled environments, sanitization methods, disposal of infected plants and monitoring of aerial conidia levels usually reduce inoculum sources, while proper heating, ventilating and lighting impede disease development since temperature below 25°C, high humidity and ultraviolet light are generally needed for conidia production and/or germination. Under field conditions, cultural practices such as rotating with non-host crops or reducing planting density and nitrogen fertilization remain the primary available tools (McPartland et al., 2000; Elad, 2016). Unfortunately, these options are not always effective and consequently, gray mold management on cannabis is very difficult, both in indoor and outdoor settings. In this regard, the use of naturally occurring beneficial microorganisms as biocontrol agents to control gray mold represents a promising alternative in cannabis, potentially also bringing added benefits such as plant growth promotion and/or biochemical traits improvement (Vujanovic et al., 2020).

In general, beneficial soil-inhabiting rhizobacteria can be effective biocontrol agents by locally repressing soil-borne pathogens by antibiosis or competition, and/or by eliciting systemic plant defenses, a phenomenon known as Induced Systemic Resistance (ISR) (Beneduzi et al., 2012). Plants can also enhance their systemic defenses in response to an earlier exposure to a pathogen, a phenomenon known as Systemic Acquired Resistance (SAR). While SAR usually induces a direct activation of defense genes expression in uninfected organs remotely from the infection site, ISR is rather associated with enhanced transcriptional changes that only become apparent after a pathogen attack (priming) (Pieterse et al., 2014). SAR is mostly effective against biotrophic pathogens susceptible to salicylic acid (SA)-mediated defenses, whereas ISR is mostly effective against necrotrophic pathogens susceptible to jasmonate (JA)- and ethylene (ET)-mediated defenses (Glazebrook, 2005). Notably, the SA- and JA/ET-pathways are often considered mutually antagonistic, even though synergistic or neutral interactions are also reported (Pieterse et al., 2009). This negative cross-talk is potentially exploited by the necrotrophic pathogen *B. cinerea* which deliberately triggers the SA-pathway to circumvent the plant defensive JA/ET-pathway (El Oirdi et al., 2011) and to benefit from SA-mediated programmed cell death (Govrin and Levine, 2000). By reinforcing JA/ET-mediated defenses through ISR elicitation, some beneficial soil-inhabiting rhizobacteria are therefore perfect candidate biocontrol agents to help protect crops against gray mold, as supported by recent reviews of successful studies and the development of commercial biopesticides (Haidar et al., 2016; Nicot et al., 2016). However, to our knowledge, the ability of beneficial bacteria to act as ISR-eliciting biocontrol agents has never been investigated in *C. sativa*, nor has the SAR elicitation by pathogens. This is probably mainly due to legal constraints and/or the unavailability of validated gene targets to detect defense responses specifically and reliably in cannabis. These research opportunities were precisely highlighted in many recent reviews on *C. sativa* (Backer et al., 2019b; Lyu et al., 2019; Taghinasab and Jabaji, 2020; Vujanovic et al., 2020).

Among more than two dozen genera of bacteria with known biocontrol and/or plant growth promoting traits (Rodríguez-Díaz et al., 2008), *Bacillus* spp. and *Pseudomonas* spp. are probably the most studied (Compant et al., 2005; Fischer et al., 2013). Many success stories have reported their ability to act as ISR-eliciting biocontrol agents against *B. cinerea*, such as in bean (Ongena et al., 2005, 2007), tomato (Kilian et al., 2000; Ongena et al., 2007), pepper (Jiang et al., 2018), oilseed rape (Sarosh et al., 2009), grapevine (Trotel-Aziz et al., 2008; Magnin-Robert et al., 2013) and *Arabidopsis thaliana* (Nie et al., 2017) for *Bacillus* spp.; and in bean (De Meyer and Höfte, 1997; Ongena et al., 2004; Meziane et al., 2005), tomato (Audenaert et al., 2002; Meziane et al., 2005), grapevine (Trotel-Aziz et al., 2008; Verhagen et al., 2010; Magnin-Robert et al., 2013; Gruau et al., 2015) and *A. thaliana* (Van der Ent et al., 2008) for *Pseudomonas* spp. Moreover, it has been suggested that combining several microorganisms in consortia can improve biocontrol effectiveness compared to using a given microorganism alone (Sarma et al., 2015). For example, consortia of *Bacillus* spp. with *Pseudomonas* spp. have already been used against *B. cinerea* in vineyards (Magnin-Robert et al., 2013), against *Fusarium udum* in pigeon pea (Dutta et al., 2008), against *F. solani* in chili (Sundaramoorthy et al., 2012), against *F. oxysporum* in banana (Akila et al., 2011), against *Verticillium dahliae* in olive (Gómez-Lama Cabanás et al., 2018), against *Sclerotinia sclerotiorum* in pea (Jain et al., 2015), against necrosis virus in sunflower (Srinivasan and Mathivanan, 2009), against *Podosphaera fusca* in melon (García-Gutiérrez et al., 2012), against *Alternaria solani* in tomato (Sundaramoorthy and Balabaskar, 2012), and to promote yield or growth of sweet cherry (Esitken et al., 2006), sunflower (Srinivasan and Mathivanan, 2009), strawberry (Pırlak and Köse, 2009), tomato (Sundaramoorthy and Balabaskar, 2012), chili (Sundaramoorthy et al., 2012) and wheat (Ansari and Ahmad, 2019). In *C. sativa*, it has been reported that consortia of bacteria not affiliated with *Bacillus* spp. or *Pseudomonas* spp. could improve the yield of hemp and marijuana cultivars (Conant et al., 2017; Pagnani et al., 2018), while one *Pseudomonas* strain reduced broomrape (parasitic weed) infestation in hemp cultivars (Gonsior et al., 2004).

In this study, we assessed the biocontrol ability of four strains of beneficial rhizobacteria to reduce gray mold symptoms on cannabis leaves. The two Gram-negative *Pseudomonas* spp. under study were the model ISR-eliciting *P. simiae* WCS417r, which controls several diseases in various plants (Berendsen et al., 2015), and *P. synxantha* LBUM223, which contributes to common scab and late blight control in potato via antibiotic production (Arseneault et al., 2014). The two Gram-positive *Bacillus* spp. under study were *B. velezensis* LBUM279 and *B. subtilis* LBUM979, which both promote cannabis growth and produce antibiotics. Each strain was applied to cannabis roots as a single treatment or in consortium treatments of each *Bacillus* sp. combined with each *Pseudomonas* sp. To further assess whether cannabis leaves express systemic immune responses, either triggered by *B. cinerea* remotely from its infection site (SAR) or elicited by the beneficial rhizobacteria (ISR), we developed new primers for Reverse Transcription quantitative Polymerase Chain Reaction (RT-qPCR) assays. The expression stability of seven *C. sativa* candidate reference genes (*TIP41*, *APT1*, *AP2M*, *EF1A*, *YLS8*, *MON1*, and *DRH1*) was first assessed. Subsequently, the expression of eight putative defense genes, reported to be mediated either by the SA-pathway (*PR1*, *PR2*, *PR5*, and *NPR1*) or the JA/ET-pathway (*LOX5*, *ERF1*, *HEL*, *PAL*), was investigated in uninfected plants and in diseased plants, primed or not by rhizobacteria. Sampling was implemented at 3 times, namely at 2, 4, and 7 days after *B. cinerea* infection.

## Materials and Methods

### Bacterial Strains and Growth Conditions

The beneficial rhizobacteria used in this study included four strains. Two *Bacillus* spp. and one *Pseudomonas* sp., namely *B. velezensis* LBUM279, *B. subtilis* LBUM979 and *P. synxantha* LBUM223, were isolated from strawberry rhizosphere soil samples collected in Bouctouche, New Brunswick, Canada (Paulin et al., 2009). Model ISR-eliciting *P. simiae* WCS417r was previously isolated from wheat rhizosphere and kindly provided by C.M.J. Pieterse (University of Utrecht, the Netherlands). All *Pseudomonas* spp. and *Bacillus* spp. were routinely grown in tryptic soy broth (TSB) (BD Difco, United States) with shaking at 120 rpm at 25°C and 37°C, respectively, until they reached their exponential growth phase. The bacterial populations were estimated by spectrophotometer readings at 600 nm and diluted to 10^8^ CFU/mL using standard curves.

### Fungal Isolate and Growth Conditions

A pathogenic strain of *B. cinerea* isolated from diseased *C. sativa* plants was obtained from Z.K. Punja (Simon Fraser University, Canada) and routinely grown at 25°C on Potato Dextrose Agar (PDA) (BD Difco, United States). Cultures were incubated under light to induce sporulation, then scraped with water to harvest conidia as previously described (Gruau et al., 2016). Conidia concentration was measured with a hemocytometer and diluted to 10^3^ conidia/mL in a solution of 0.067 M KH_2_PO_4_ and 0.11 M glucose to promote infection (Van Den Heuvel, 1981).

### Cannabis Growth Chamber Experiments

Cannabis seeds (*C. sativa* hemp cultivar Anka) obtained from Valley Bio Limited (Cobden, Canada) were sown in a mixture of peat-based growing medium and vermiculite (75-25% v/v) (Premier Tech, Rivière-du-Loup, Canada) in a growth chamber under a 18/6 h day/night photoperiod (photo flux density of 300 μmol m^–2^ s^–1^), at 23°C and 70% relative humidity. After one week, 216 seedlings at a similar growth stage were transplanted into individual 4-inch diameter pots and were randomly assigned to receive bacteria-priming root treatments as follows: 24 plants received one of the four single-bacteria treatments (totalizing 96 plants) by inoculating 10 mL of the corresponding standardized bacterial culture and 10 mL of tap water into the soil; 24 plants received one of the four consortium treatments (four combinations of each *Bacillus* sp. with each *Pseudomonas* sp., totalizing 96 plants) by inoculating 10 mL of both corresponding standardized bacterial cultures; and 24 control plants were mock-primed by inoculating 20 mL of tap water.

One week after receiving one of the bacteria-priming root-treatments, half the plants were kept healthy (108 uninfected plants), while the other half were infected with *B. cinerea* by depositing a 10 μL droplet of standardized conidial suspension on each side of the central vein of a main leaflet from the second true leaves pair (108 diseased plants). All plants were then enclosed in clear plastic bags to keep humidity high, using the same temperature and photoperiod as described above.

**FIGURE 1:**
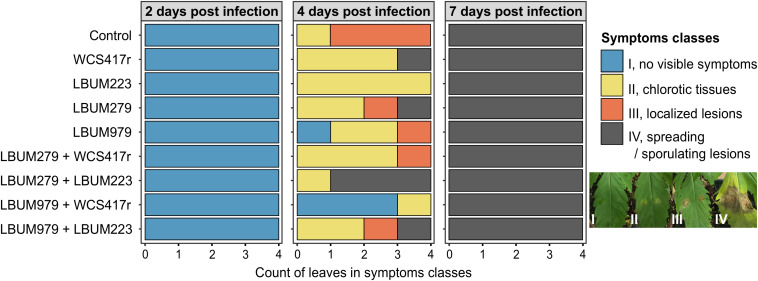
Symptoms severity on cannabis leaves 2, 4, and 7 days after infection by *B. cinerea*. Cannabis seedlings were primed with single-bacteria root treatments (*Pseudomonas* strains WCS417r and LBUM223; *Bacillus* strains LBUM279 and LBUM979), or consortium root treatments (LBUM279 + WCS417r, LBUM279 + LBUM223, LBUM979 + WCS417r, LBUM979 + LBUM223), or mock-primed with water (control). After one week, plants were infected with *B. cinerea* (2 droplets containing 10^3^ conidia/mL) and kept under high humidity for 2, 4, and 7 days. Symptoms were assigned into 4 severity classes: I, no visible symptoms (blue); II, chlorotic tissues forming a yellow halo (yellow); III, necrotic localized lesions smaller than the original droplets size (orange); IV, large spreading lesions with tissue maceration expanding beyond the original droplets and/or sporulating (gray). Stacked barplots represent the number of leaves assigned into each class per bacterial treatment, with 4 independent biological replicates at each harvest time (each leaf comes from a different plant). No statistically significant biocontrol protection was reported when comparing bacteria-treated plants to control plants, at each harvest time (Kruskal-Wallis rank sum test with Dunn pairwise comparisons and Benjamini-Hochberg correction, α = 0.05).

After 2, 4, and 7 days (days post infection, dpi), symptoms were recorded and samples for gene expression analysis were harvested (destructive harvest). There were 4 biological replicates (4 different plants) from each group harvested at each time. A symptoms severity class was assigned to the infected leaf of each plant as indicated in Figure 1. For the downstream RT-qPCR assays, one infected leaf (designated thereafter as a local leaf) and one leaf opposite from the infection site (designated thereafter as a systemic leaf) from each diseased plant were harvested, as well as one leaf from each uninfected plant (designated thereafter as an uninfected leaf). A total of 324 samples were thus collected. About 30 mg of tissues (about 1 cm^2^), either from healthy tissues or from tissues surrounding a lesion, were immediately frozen in liquid nitrogen until RNA extraction.

To assess the ability of the bacteria to colonize the rhizosphere and to promote cannabis growth, 35 one-week old cannabis seedlings were grown and transplanted as described above, then received one of the four single-bacterial treatments or the water mock-treatment (control), totalizing 7 biological replicates per treatment. All plants were grown for 3 weeks under the same temperature and photoperiod as described above, then whole plants were harvested and fully dried at 70°C for 5 days before measuring the total dry weight of each plant (Supplementary Figure S1).

### Primers Design

Homologous mRNA sequences corresponding to eight known defense genes (four associated with the JA/ET-pathway and four associated with the SA-pathway) and seven reference genes (Table 1), were retrieved from GenBank (National Center for Biotechnology Information, United States) for plants as closely related to cannabis as possible. Those sequences were aligned against a *C. sativa* cultivar Purple Kush transcriptome assembly (Van Bakel et al., 2011) using BioEdit Sequence Alignment Editor version 7.0.5.3 (Hall, 1999). Best matching fragments were translated into protein sequences to predict functional domains using InterPro (Table 1). A fragment encoding an important molecular function for each gene was then used as target on which PCR primers were designed by Primer Express v. 3.0.1 (Thermo Fisher Scientific, United States) with the following parameters: primer melting temperature (TM) 58°C-60°C, optimal primer length 20 bp, amplicon maximum TM 85°C, and amplicon length 50 bp-150 bp (Table 2). Primer specificity was first assessed using Primer-BLAST (National Center for Biotechnology Information, United States), then validated by melting-curve analyses on cannabis cDNA using qPCR (Supplementary Figure S2).

**TABLE 1:** Description of cannabis putative defense genes and candidate reference genes.

Symbol	Gene name	Gene predicted function	InterPro domain	Accession No.	Length (bp)
**Defense genes: JA/ET-pathway**					
*LOX5*	linoleate 9S-lipoxygenase 5	oxylipins biosynthesis	IPR020833	XM_030648324	2898
*ERF1*	ethylene response factor 1	AP2/ERF transcription factor	IPR001471	XM_030652454	718
*HEL*	hevein-like protein	antifungal chitin-binding protein	IPR001153	XM_030650355	921
*PAL*	phenylalanine ammonia-lyase	phenylpropanoids biosynthesis	IPR022313	XM_030639598	2227
**Defense genes: SA-pathway**					
*PR1*	pathogenesis-related protein 1	antifungal protein	IPR001283	XM_030633258	698
*PR2*	pathogenesis-related protein 2	basic β-1,3-glucanase	PS00587	XM_030638432	1228
*PR5*	pathogenesis-related protein 5	thaumatin-like protein	IPR017949	XM_030630229	1433
*NPR1*	non-expressor of pathogenesis-related genes 1	transcription co-activator of PR genes	IPR021094	XM_030624173	2966
**Reference genes**					
*TIP41*	TAP42 interacting protein of 41 kDa	Target-of-Rapamycin (TOR) pathway	IPR007303	XM_030630502	1144
*APT1*	adenine phosphoribosyl transferase 1	nucleobases salvage	IPR000836	XM_030642128	1193
*AP2M*	adaptor protein-2 mu-adaptin	clathrin-dependent endocytosis	IPR018240	XM_030645277	1898
*EF1A*	elongation factor 1-α	proteins translation	IPR004161	XM_030651612	1790
*YLS8*	yellow leaf specific protein 8	pre-mRNA splicing	IPR004123	XM_030646115	825
*MON1*	monensin sensitivity 1	vacuolar trafficking	IPR004353	XM_030648523	2311
*DRH1*	DEAD box RNA helicase 1	RNA metabolism	IPR000629	XM_030627008	2839

*Annotations, accession numbers, and mRNA lengths were retrieved from GenBank. Predicted protein functional domains were identified by InterPro and corresponding coding sequences were used for primers design.*

**TABLE 2:** qPCR primers designed in this study.

Gene	Forward primer sequence (5′-3′)	Reverse primer sequence (5′-3′)	Amplicon size (bp)	Amplicon TM (°C)	E (%)	R^2^
**Defense genes: JA/ET-pathway**						
*LOX5*	GCATGCTGTGATTGAGCCTTT	GTAGATTGGGTGGAGAACACTTAGC	67	76.0	90.0	0.966
*ERF1*	CGGCCGAAATTAGGGATTC	ATCAAATGTTCCAAGCCAAACTC	59	75.0	114.6	0.994
*HEL*	CATGGCGCAGCAAATATGG	CCCCTAGGTCCGGATGGT	55	78.5	115.1	0.996
*PAL*	ACAACGTCACCCCATGCTTAC	GTACAAGGTCACCGGATGCA	60	77.0	111.9	0.990
**Defense genes: SA-pathway**						
*PR1*	GCGTAACTCGGTTCGTTTGG	TGCAAGTGATGAAGGTACCCTTATT	71	77.5	103.6	0.994
*PR2*	TTCGTTGGAGATTGTTGTTTCG	CTCAAACGACGTCGCTGTTC	67	80.5	104.5	0.996
*PR5*	GGTTGCACCTTCGACAATTCA	TGACCGGAACCGCAGTCT	62	78.5	114.6	0.990
*NPR1*	AAGAGAGATGTGGAGAAATCCAATG	CAGCCATCGTATGCAAAGACA	62	76.0	114.3	0.990
**Reference genes**						
*TIP41*	GGCACCCAAAGAGCCTATTCT	CCCCATTATCTGCAAGTTCATCT	71	73.0	106.4	0.992
*APT1*	TTGCAACTGGAGGAACCTTGT	CATCCACTCCAACACGTTCAA	60	77.0	108.9	0.992
*AP2M*	CAAGTTACGGGTGCTGTTGGT	CACAATATCCAAAAACACCTCATTCT	75	76.0	-	-
*EF1A*	TGCTCCCACCGGTCTGA	GCCTCGTGGTGCATCTCAA	54	78.5	-	-
*YLS8*	GATGGATGAAGTTTTGGCATCA	TCCACAAGGTATATCACAGCAAAGTT	66	74.0	-	-
*MON1*	GCTAGCAGGATTTTCAGCAACA	CACGATCCCCTCCATTCTCA	65	74.0	-	-
*DRH1*	TCGAATGCTTGACATGGGTTT	GCGAGTAGGCACCTCCTTCA	64	75.5	-	-

*Primer sequences and predicted amplicon sizes were provided by Primer Express v. 3.0.1. Amplicons melting temperature (TM), qPCR amplification efficiency (E) and regression coefficient (R^2^) were calculated by Bio-Rad CFX Manager software using cannabis cDNA. Values indicated by (-) were not estimated.*

### RNA Extraction and RT-qPCR Assays

Total RNA was extracted from cannabis leaf samples using the RNeasy Plant Mini kit (Qiagen, Germany) and a TissueLyser, following manufacturer’s instructions. The optional on-column DNase treatment was performed and followed by two additional DNA digestion steps using the Turbo DNA-free kit (Thermo Fisher Scientific, United States). Satisfactory elimination of genomic DNA was validated by confirming absence of qPCR amplification on a set of representative samples for which the RT step was omitted (no-RT control). RNA samples concentration was measured with a Qubit RNA BR assay (Thermo Fisher Scientific, United States) and diluted to a final concentration of 100 ng/μL prior to RT. Synthesis of 110 μL of cDNA was performed using the TaqMan Reverse Transcription Reagent kit with the OligodT_16_ primer (Thermo Fisher Scientific, United States) at 48°C for 30 min followed by a 5 min inactivation at 95°C. Resulting cDNA was used for 10 μL qPCR reactions in a CFX96 Real Time PCR Detection System (Bio-Rad, United States) containing 5 μL of iTaq Universal SYBR Green Supermix (Bio-Rad, United States), 2 μL of cDNA template, 1 μL of water, and 1 μL of each primer (5 μM). No-template controls (NTC) were included using water instead of cDNA. All qPCR reactions were conducted with 3 technical replicates and the following protocol: 95°C for 3 min, 40 cycles at 95°C for 10 s, 60°C for 30 s, followed by a melting curve from 65 to 95°C to confirm primer specificity. RT-qPCR data were analyzed with qbase^plus^ version 3.2 (Biogazelle, Belgium). Samples with an average quantification cycle (Cq) value superior to 35 were excluded from the corresponding target analysis to prevent bias from any residual genomic DNA. Inter-plate variation was removed by appointing an Inter-Run Calibrator (IRC) consisting of an identical cDNA sample on all plates.

### Reference Genes Expression Stability Pilot Study

**FIGURE 2:**
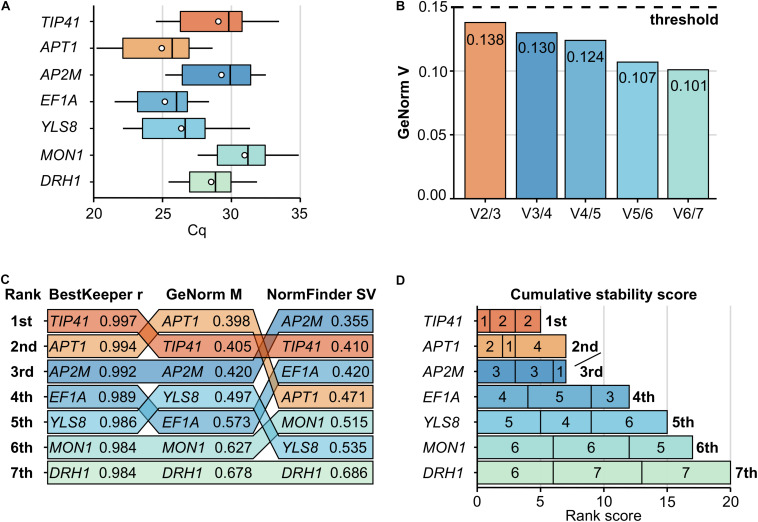
Determination of the optimal set of reference genes during a pilot study. The expression stability of seven candidate genes (*TIP41*, *APT1*, *AP2M*, *EF1A*, *YLS8*, *MON1*, and *DRH1*) was assessed during an independent pilot study. Samples harvest, total RNA extraction and RT-qPCR assays were performed similarly to the defense genes expression study, with 20 biological replicates and 3 technical replicates. **(A)** Cq values distribution per gene are presented on boxplots with the interquartile range as a box, the lowest and highest values as whiskers, the median as an inner line and the mean as a dot. **(B)** The determination of the optimal number of reference genes is based on GeNorm pairwise variation values (V_n/n + 1_). As V_2/3_ (highlighted in orange) dropped below the commonly used 0.15 threshold (dashed line), two reference genes should suffice. **(C)** Expression stability values from BestKeeper (r), GeNorm (M) and NormFinder (SV) are presented for each gene, ranked from the most stable gene (1st, top) to the least stable (7th, bottom). Lower GeNorm and NormFinder values and higher BestKeeper value indicate more stable expression. **(D)** The final determination of the most stable reference genes is based on their low cumulative stability score, which is the sum of their ranks obtained from the three methods used (rank values inside bars, from left to right: BestKeeper, GeNorm, and NormFinder). The optimal set of reference genes (a pair formed by *TIP41* and *APT1*) is highlighted in orange across all panels.

The expression stability of seven candidate reference genes (*TIP41*, *APT1*, *AP2M*, *EF1A*, *YLS8*, *MON1*, and *DRH1*) was assessed during a pilot study on 20 representative samples independent from the 324 samples for the main experiment. Samples harvest, RNA extraction, cDNA synthesis and qPCR with 3 technical replicates were performed as described above. To illustrate the overall variability of transcripts levels, the Cq values distribution per gene was illustrated on boxplots (R version 3.5.2, package ggplot2) (Figure 2A).

To confidently select a set of stable reference genes among the seven candidates, the Cq data were analyzed by three different software methods. GeNorm (Vandesompele et al., 2002) was accessed within qbase^plus^ version 3.2 (Biogazelle, Belgium), while NormFinder (Andersen et al., 2004) and BestKeeper (Pfaffl et al., 2004) were accessed through the web-based tool RefFinder. GeNorm generated the average pairwise expression ratio (M) for each gene, which was negatively correlated with its stability. Similarly, the NormFinder software provided a stability value (SV) for each gene, which was negatively correlated with its stability. Conversely, the BestKeeper software generated a coefficient (r) reflecting the Pearson correlation between each gene and an index based on the other reference genes, which was positively correlated with the gene stability. All three methods ranked all seven genes from 1 to 7 (from best/most stable to worst/least stable, respectively) (Figure 2C). To integrate these results, a cumulative stability score was established for each gene by summing its rank within the overall ranking order established by each software. A gene cumulative stability score was negatively correlated with its stability (Figure 2D).

Finally, the optimal number of reference genes required for adequate expression normalization was determined by GeNorm. The GeNorm (V_n/n + 1_) values represented the reduction in pairwise variation achieved by using (n + 1) reference genes instead of (n) during the process of expression normalization. A value below 0.15 indicated that no more genes than (n) was required (Vandesompele et al., 2002; Figure 2B).

### Defense Genes Expression Study

**FIGURE 3:**
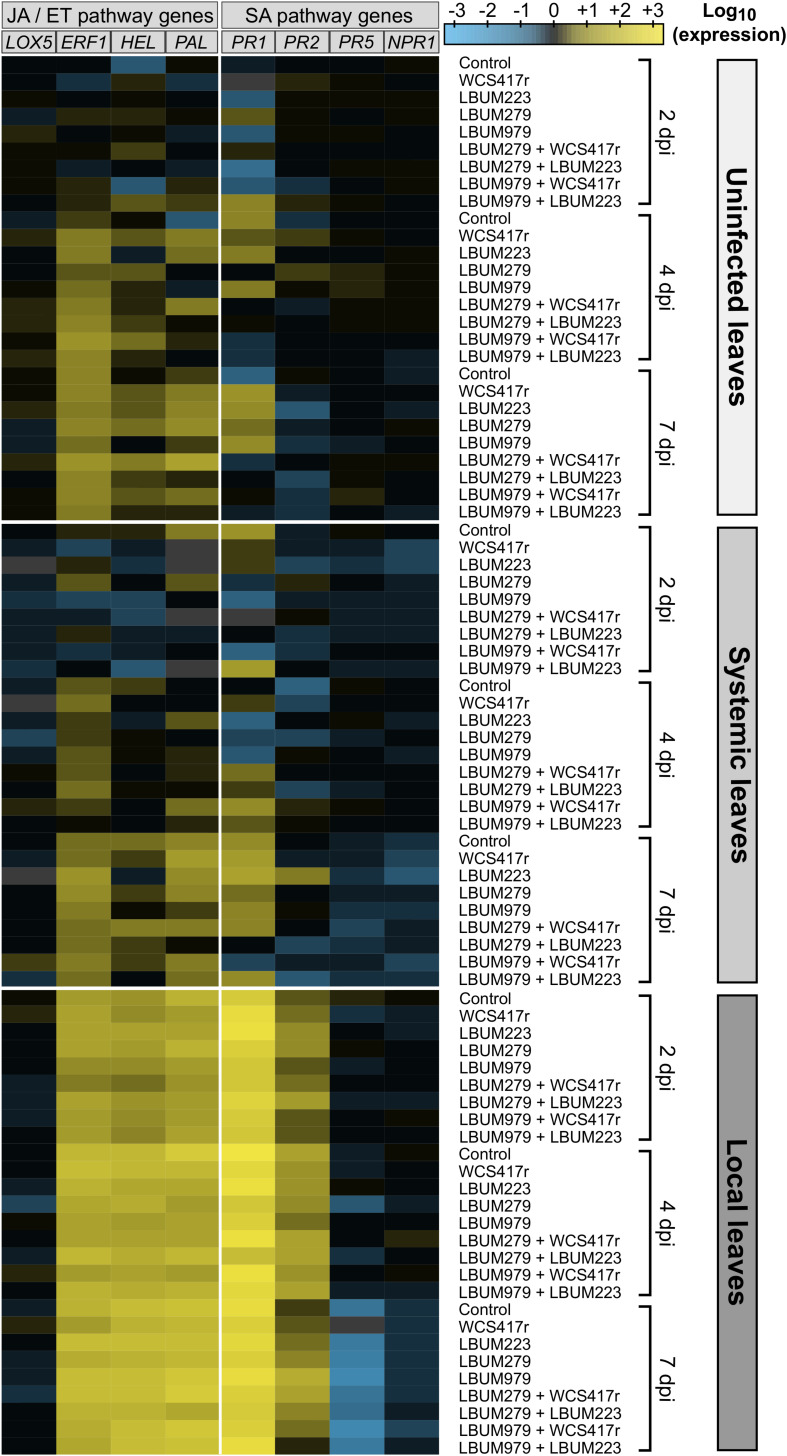
Heatmap showing the expression pattern of putative JA/ET- and SA-mediated genes in cannabis plants primed by *Pseudomonas* and/or *Bacillus* strains and infected by *B. cinerea*. Cannabis seedlings were primed with one of four single-bacteria root treatments, or one of four consortium root treatments, or mock-primed with water (control). After one week, plants were infected with *B. cinerea* (diseased plants) or left untreated (uninfected plants). After 2, 4, and 7 days (2, 4, and 7 dpi), leaf tissues were sampled from uninfected plants (uninfected leaves) and from diseased plants (systemic leaves remote from infection site and local infected leaves). Total RNA was extracted and the expression level of eight putative defense genes was analyzed by RT-qPCR accounting for primer amplification efficiency. Data were normalized with the reference genes *TIP41* and *APT1*. Fold changes of expression were scaled per gene relatively to the average expression in uninfected leaves at 2 dpi (set as black on color scale). The heatmap represents the log_10_-transformed mean expression from 4 independent biological replicates and 3 technical replicates; blue indicates lower gene expression than the average expression in uninfected leaves at 2 dpi (downregulation) while yellow indicates higher gene expression (upregulation). Missing values are grayed out.

RNA extraction and RT-qPCR were performed as described above with 3 qPCR technical replicates for the 324 leaf samples harvested during the growth chamber experiment. Fold changes of relative expression of eight putative defense genes (*LOX5*, *ERF1*, *HEL*, *PAL*, *PR1*, *PR2*, *PR5*, and *NPR1*) were estimated by normalizing data using the two most stable reference genes identified during the pilot study (*APT1* and *TIP41*) and by scaling expression relatively to the average expression in uninfected plants at first harvest (2 dpi) (therefore arbitrarily set to a level of 1). The amplification efficiency and regression coefficient (R^2^) for each of the 10 primer pairs were determined by standard curves generated from cDNA 2-fold dilution series (1, 1/2, 1/4, 1/8, 1/16, 1/32) and accounted for during the fold changes calculations (Table 2). All data were log_10_-transformed before generating a heatmap with the web-based tool Heatmapper (Babicki et al., 2016; Figure 3) and performing statistical analyses.

### Statistical Analyses

For disease symptoms recorded during the growth chamber experiment, a Kruskal-Wallis rank sum test assessed whether proportions of symptoms severity classes differed statistically between groups of plants primed by different bacterial treatments, at each harvest time (4 biological replicates, α = 0.05) (Figure 1). Any significant difference was followed by Dunn pairwise comparisons with Benjamini-Hochberg correction to identify which groups were different from the water-treated group.

For cannabis plant growth promotion, a Kruskal-Wallis rank sum test followed by Dunn pairwise comparisons with Benjamini-Hochberg correction assessed whether dry weight from plants treated with bacteria differed from control plants (7 biological replicates, α = 0.05) (Supplementary Figure S1). All statistical analyses were performed in R version 3.5.2 (package FSA).

**FIGURE 4:**
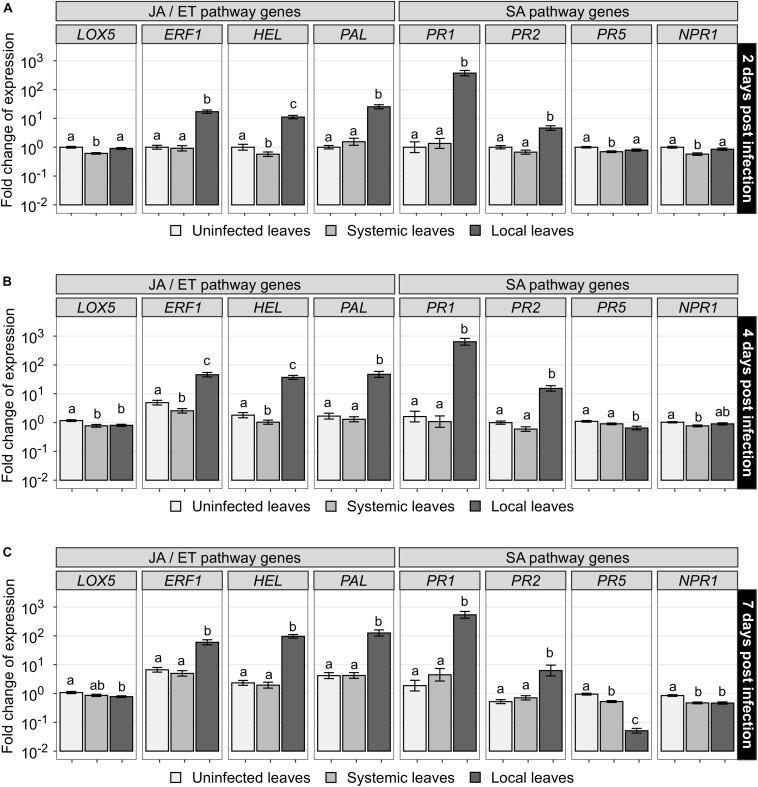
Effects of *B. cinerea* treatment on the expression of putative JA/ET- and SA-mediated genes at different harvest times in cannabis leaves. Fourteen-day-old cannabis plants were infected with *B. cinerea* (diseased plants) or left untreated (uninfected plants). Leaf tissues were sampled after **(A)** 2 days, **(B)** 4 days, and **(C)** 7 days. Total RNA was extracted and the expression level of eight putative defense genes was analyzed by RT-qPCR in uninfected plants (uninfected leaves, white) and diseased plants (systemic leaves remote from infection site, light gray, and local infected leaves, dark gray). Fold changes of expression were scaled per gene relatively to the average expression in uninfected leaves at 2 days after infection (expression level of 1). Means and standard errors of 36 independent biological replicates and 3 technical replicates are displayed on a log_10_ scale. Treatments sharing the same letter are not significantly different according to Wald chi-square test with Benjamini-Hochberg correction, α = 0.05.

For fold changes of normalized relative expression of the eight defense genes across 324 samples from the ISR/SAR expression study, log_10_-transformed data from 3 qPCR technical replicates were analyzed with a three-way mixed ANOVA model, sum of squares type II and Wald *F* test with Kenward-Roger approximation for degrees of freedom (4 biological replicates, α = 0.05). The three fixed factors were the time of harvest (3 levels: 2, 4, and 7 dpi), the pathogen treatment (3 levels: local leaves, systemic leaves, uninfected leaves), and the bacterial treatment (9 levels: 4 single-bacteria treatments, 4 consortium treatments, 1 water mock-treatment), while a random factor was added to account for pairing of systemic and local leaves harvested from the same plant. As the interaction between all three factors was not significant, it was removed from the model to deal with missing values. *Post hoc* multiple comparisons with Wald chi-square test and Benjamini-Hochberg correction were used to investigate the effect of significant factors and the effect of one factor within each level of the other factor in the case of a significant interaction between two factors (Figure 4, Supplementary Figures S3, S4, Supplementary Table S1). All statistical analyses were performed in R version 3.5.2 (packages lme4, car and phia).

## Results

### Treatment With Beneficial Rhizobacteria Does Not Significantly Reduce Gray Mold Symptoms but Some Promote Plant Growth

To assess the biocontrol ability of four beneficial rhizobacteria against *B. cinerea*, young cannabis plants were sown in a growth chamber and received different bacteria-priming root treatments. One week later, one leaf was infected with a solution of conidia and symptoms were recorded after 2, 4, and 7 days using a 4-class severity scale (Figure 1). On the first day of observation (2 dpi), no symptoms were yet visible on any plants (severity class I). However, 4 days after infection (4 dpi), noticeable symptoms had developed at the droplet locations on most infected leaves, displaying yellowed chlorotic halos (severity class II), or necrotic localized lesions (severity class III), or spreading lesions with tissue maceration and/or secondary sporulation (severity class IV). Overall, plants primed with LBUM979 + WCS417r or with LBUM223 were relatively exempt of symptoms, displaying only chlorotic halos at most, while control plants and plants primed with LBUM279 + LBUM223 displayed the strongest symptoms with most infected leaves covered by lesions. However, perhaps in part due to the small sample size, no statistically significant biocontrol protection was reported between bacteria-treated plants and control plants (Kruskal-Wallis rank sum test with Dunn pairwise comparisons and Benjamini-Hochberg correction, α = 0.05). On the last day of observation (7 dpi), all infected leaves were equally covered by spreading and/or sporulating lesions (severity class IV), regardless of the bacterial treatment. This latter result clearly suggests that priming cannabis plants with the bacteria did not significantly improve long-term resistance to gray mold in the conditions tested. However, under the same conditions, inoculating cannabis seedlings with the bacteria resulted in an increased dry weight for most plants compared to control, after 3 weeks of growth (Supplementary Figure S1). The plant growth promoting effect was more pronounced with the *Bacillus* strains than with the *Pseudomonas* strains and was statistically significant with LBUM979 (Kruskal-Wallis rank sum test with Dunn pairwise comparisons and Benjamini-Hochberg correction, α = 0.05).

### Primer Specificity and qPCR Amplification Efficiency

To investigate potential defense responses in cannabis leaves at the molecular level, new RT-qPCR primers were designed to monitor the expression of eight putative defense genes and seven candidate reference genes (Table 1). Newly designed primers are listed in Table 2 with relevant information. A single PCR amplicon was produced by each primer pair with a single TM peak on the melting curve (Supplementary Figure S2), confirming the specific amplification of each gene. The amplification efficiencies, estimated for all eight defense genes and the two selected reference genes, ranged from 103.6% to 115.1%, apart from *LOX5* at 90.0%. The correlation coefficients were all superior or equal to 0.99, supporting standard curves reliability (Table 2).

### TIP41 and APT1 Are the Most Appropriate Reference Genes

To normalize defense genes expression data from the RT-qPCR assays, a set of appropriate reference genes had to be selected first. Therefore, the expression stability of seven *C. sativa* candidate reference genes (*TIP41*, *APT1*, *AP2M*, *EF1A*, *YLS8*, *MON1*, and *DRH1*) was assessed during a pilot study (Table 1). Transcripts levels of the seven candidates exhibited a broad range of Cq values across the 20 samples (Figure 2A). Based on their mean Cq, *APT1* (Cq 24.94) and *MON1* (Cq 30.96) were the highest and lowest expressed genes respectively, with *APT1* being almost sixty-five times more expressed than *MON1* (Figure 2A).

The stability ranking of the seven candidates during the pilot study was established by BestKeeper, GeNorm, and NormFinder analyses (Figure 2C). According to BestKeeper, *TIP41* (r 0.997) and *APT1* (r 0.994) were ranked the most stable genes, while *DRH1* (r 0.984) and *MON1* (r 0.984) were tied as the least stable genes. According to GeNorm, *APT1* (M 0.398) and *TIP41* (M 0.405) were again ranked the most stable genes, while *MON1* (M 0.627) and *DRH1* (M 0.678) were the least stable genes. According to NormFinder, *AP2M* (SV 0.355) and *TIP41* (SV 0.410) were ranked the most stable genes, while *YLS8* (SV 0.535) and *DRH1* (SV 0.686) were the least stable genes.

The cumulative stability score integrated the results from the three above methods, by summing the ranks obtained for each gene. The genes with the smallest cumulative scores were considered the most stable. The final recommendations are listed in Figure 2D. *TIP41* (cumulative score 5) was ranked as the most stably expressed gene, followed by *APT1* and *AP2M* tied for second and third places (cumulative score 7), while *MON1* (cumulative score 17) and *DRH1* (cumulative score 20) were the least stably expressed genes.

Finally, the pairwise variation values (V_n/n + 1_) provided by GeNorm were used to determine the optimal number of reference genes suitable for expression normalization (Figure 2B). As the first pairwise variation value dropped below the 0.15 threshold (V_2__/__3_ 0.138), it indicated that two reference genes should suffice. Moreover, a visual interpretation of the pairwise variations trend suggested that adding a third reference gene would not greatly reduce the pairwise variation (V_3__/__4_ 0.130).

Based on the ranking from the three software methods, the cumulative stability scores, and the GeNorm V values, it was concluded that *TIP41* and *APT1* constituted an optimal set of reference genes to properly normalize subsequent RT-qPCR data.

### Defense Genes Expression Study

To investigate whether cannabis defenses were triggered locally and/or systemically by the pathogen *B. cinerea* and/or the beneficial rhizobacteria, the expression of eight putative defense genes, predicted to be mediated either by the SA-pathway (*PR1*, *PR2*, *PR5*, *NPR1*) or the JA/ET-pathway (*LOX5*, *ERF1*, *HEL*, *PAL*) (Table 1), was monitored over time by RT-qPCR in young cannabis plants primed by the bacteria and infected one week later by the pathogen. The log_10_-transformed fold changes results are summarized on a heatmap to overview the general pattern of genes relative expression (Figure 3). Expression of most genes clearly appeared to be upregulated in local leaves compared to uninfected leaves and systemic leaves (away from the infection site), regardless of the pathway the genes presumably belonged to. Statistical analyses, using a three-way mixed ANOVA model, are detailed below to further examine the effect of harvest time (Supplementary Figure S3, Supplementary Table S1), pathogen treatment (Figure 4, Supplementary Table S1), and bacteria-priming treatment (Supplementary Figure S4) on genes expression.

### Infection by *B. cinerea* Strongly Upregulates the Expression of *ERF1*, *HEL*, *PAL*, *PR1* and *PR2* Locally

First, RT-qPCR analyses revealed that the expression of most genes varied depending on the harvest time (Supplementary Figure S3, Supplementary Table S1). However, since similar trends occurred similarly in all types of leaves, including the uninfected ones, these variations are likely due to basal plant growth and maturation over time rather than induced by the pathogen attack. Next, the genes expression level in local leaves was compared to their expression level in uninfected leaves at corresponding harvest time to reveal the local response to *B. cinerea* infection (Figure 4, Supplementary Table S1). The expression of *ERF1*, *HEL*, *PAL*, *PR1*, and *PR2* was found to be significantly upregulated locally by the pathogen attack and this effect was sustained during the whole week following infection. The net fold change variations (ratios of expression in local leaves to uninfected leaves, for each corresponding harvest time) ranged from 9.0- to 17.1-fold for *ERF1*, 11.2- to 41.1-fold for *HEL*, 25.3- to 30.2-fold for *PAL*, 286.7- to 394.2-fold for *PR1*, 4.6- to 15.5-fold for *PR2*. The expression of the other genes, namely *LOX5*, *PR5* and *NPR1*, remained stable or was significantly downregulated in local leaves, with net fold change variations ranging from 0.1 to 0.9-fold (Supplementary Table S1). Altogether, these results indicate that a strong response to *B. cinerea* infection occurred locally.

### Infection by *B. cinerea* Does Not Systemically Induce Putative Defense Genes Expression

In the case of the systemic leaves, the expression of all genes remained stable or was significantly downregulated compared to uninfected leaves for *LOX5*, *ERF1*, *HEL*, *PR5*, and *NPR1* (Figure 4). The net fold change variations (ratios of expression in systemic leaves to uninfected leaves, for each corresponding harvest time) were no lower than 0.5-fold, indicating a downregulation by half at most (Supplementary Table S1). Since downregulations of only small amplitude and no significant upregulation were observed, these results suggest a lack of systemic defenses induction at the transcriptomic level in cannabis leaves following *B. cinerea* infection, in the conditions tested.

### Treatment With Beneficial Rhizobacteria Does Not Enhance Putative Defense Genes Expression

Finally, regarding the ability of the bacterial treatments to enhance the expression of cannabis putative defense genes, no statistically significant differences were found between bacteria-treated plants and water-treated plants (control), for any of the eight genes examined and regardless of the pathogen treatment (Supplementary Figure S4). Overall, all the bacteria-priming root-treatments failed to upregulate the expression of the putative defense genes in cannabis leaves, even in the presence of the pathogen.

## Discussion

### Efficient Primers Design and Stable Reference Genes in Cannabis

Gene expression analyses are widely used to elucidate the transcriptional reprogramming underlying numerous molecular mechanisms in living organisms, and plant defense pathways are no exception. The RT-qPCR has become a common technique to monitor gene expression profiles thanks to its accuracy, high-throughput potential and sensitivity (Huggett et al., 2005). However, specific primers must be designed for newly studied organisms, like *C. sativa*. In this study, 15 new PCR primer pairs were designed using the transcriptome of *C. sativa* cultivar Purple Kush (Van Bakel et al., 2011) and successfully amplified cDNA from another cultivar, Anka, with good efficiency and specificity (Table 2, Supplementary Figure S2). This success opens the door to new exciting research opportunities in cannabis.

Another crucial step to ensure RT-qPCR reliability is to properly normalize data to stably expressed reference genes, also commonly referred to as housekeeping genes (Huggett et al., 2005). To date, several studies have reported stable reference genes in *C. sativa*, such as actin, glyceraldehyde-3-phosphate dehydrogenase (*GAPDH*) and 18S ribosomal RNA (Mangeot-Peter et al., 2016; Booth et al., 2017; Guo et al., 2018). However, these genes were found to be commonly regulated and unstable under a wide range of experimental conditions (Huggett et al., 2005). Other studies have appointed cannabis reference genes for RT-qPCR assays but without validating beforehand their expression stability (Marks et al., 2009; Cascini et al., 2013; Docimo et al., 2014). Besides, none of these studies were conducted in cannabis plants under biotic stress conditions, whereas infection by a pathogen may affect which reference genes are selected, as illustrated by a study in hop infected by the fungus *Verticillium albo-atrum* (Štajner et al., 2013). In a pilot experiment, we selected seven orthologous reference genes from the last aforementioned study (Table 1), since hop is a Cannabaceae plant closely related to cannabis. Among these seven candidates, *TIP41* and *APT1* were found to be the most stably expressed under our experimental conditions (Figure 2), indicating that a combination of *TIP41* and *APT1* is suitable for normalizing RT-qPCR assays in cannabis leaves infected by *B. cinerea*. This result is supported by previous studies that determined that *TIP41* and/or *APT1* were the optimal reference genes for lettuce and tomato under abiotic or biotic stress conditions (Alfenas-Zerbini et al., 2009; Borowski et al., 2014; Lacerda et al., 2015). Our findings could serve as guidelines for future work on cannabis, but should not be blindly transposed since the optimal combination of reference genes may change under different experimental settings (Huggett et al., 2005).

### Local Elicitation of Putative Cannabis Defenses by *B. cinerea*

Plants are sessile organisms that rely on a complex innate immune system to cope with incessant stresses in their environment. Upon perception of a pathogen attack, plant inducible defenses can be activated to limit disease progression and are finely regulated by transcription factors and major phytohormones such as JA, ET and SA, creating a sophisticated signaling network of local and systemic responses (Pieterse et al., 2014). With little knowledge on *C. sativa* defensive mechanisms against pests and diseases, identifying genes differentially expressed during its interaction with pathogens could play an important role in improving crop management. Consequently, in this study, we chose eight defense-related genes (*LOX5*, *ERF1*, *HEL*, *PAL, PR1*, *PR2*, *PR5, NPR1*) that are well described markers of the JA/ET- and the SA-pathways in other plants, and monitored the expression of their homologous counterparts in cannabis leaves during gray mold disease onset (Figure 3). The expression of *ERF1*, *HEL*, *PAL*, *PR1* and *PR2* was strongly upregulated locally following infection (Figure 4, Supplementary Table S1), even though these responses were not sufficient to suppress lesions development. This effect was sustained over time, starting before symptoms appearance, and lasting after necrotic lesions complete development. This confirms that major transcriptome changes can occur during the critical phase during which the fungus begins to establish itself within plant tissues (Windram et al., 2012).

### JA/ET-mediated Putative Defenses

Defenses mediated by the JA/ET-pathway are usually effective against necrotrophic pathogens such as *B. cinerea* (Thomma et al., 2001). The *ERF1* gene was among the three putative JA/ET-mediated markers to be upregulated in cannabis local leaves infected by *B. cinerea*. ERF1 is a transcription factor in the Apetala2/Ethylene Response Factor (AP2/ERF) superfamily, one of the most important families of stress-responsive transcription factors in plants (Lu et al., 2013). In *A. thaliana*, ERF1 integrates signaling from the JA- and the ET-pathways in order to activate the transcription of many antifungal defense genes (Lorenzo et al., 2003). *ERF1* expression itself is upregulated during gray mold infection in *A. thaliana* (Berrocal-Lobo et al., 2002; AbuQamar et al., 2006), in lettuce (De Cremer et al., 2013), and in grapevine (Gruau et al., 2015), supporting the results obtained in cannabis.

A second putative JA/ET-mediated gene, predicted to encode an hevein-like HEL protein, was also highly upregulated in cannabis local leaves infected by *B. cinerea*. Comparative sequence analyses have shown previously that HEL proteins contain a barwin domain and share similarities with the antifungal protein hevein in rubber tree, the wound-induced WIN1 and WIN2 proteins in potato, and the PR4 protein in tobacco (Potter et al., 1993). A chitin-binding activity is suggested to confer its role in antifungal plant defensive mechanisms (Bertini et al., 2012). *HEL* expression is also greatly induced by *B. cinerea* infection in *A. thaliana* (AbuQamar et al., 2006), supporting the results obtained in cannabis.

Finally, the third significantly upregulated putative JA/ET-mediated marker in cannabis infected local leaves, *PAL*, likely encodes a phenylalanine ammonia-lyase which catalyzes the first step in the biosynthesis of phenylpropanoids. Phenylpropanoids are precursors to complex secondary metabolites such as flavonoid pigments, antimicrobial phytoalexins and lignin that reinforces cell walls. *PAL* has been one of the first identified plant defense genes and is induced by fungal elicitors and/or fungal pathogens in bean, parsley, pea, potato, *A. thaliana* (Wanner et al., 1995), and more recently by *B. cinerea* in grapevine (Bézier et al., 2002; Gruau et al., 2015) and lettuce (De Cremer et al., 2013), supporting the results obtained in cannabis.

Surprisingly, the *LOX5* gene, predicted to encode a 9S-lipoxygenase (9S-LOX), was the only putative marker from the JA/ET-pathway to be downregulated following *B. cinerea* infection. LOX are iron-containing enzymes that catalyze the dioxygenation of polyunsaturated fatty acids to produce a variety of metabolites, collectively called oxylipins (Vellosillo et al., 2007). Oxylipins yielded by 9S-LOX are distinct from those yielded by 13S-LOX and even though their physiological roles are not completely elucidated, some have been demonstrated to act as inducible plant defensive mechanisms against (hemi)biotrophic pathogens in potato (Weber et al., 1999; Kolomiets et al., 2000), tobacco (Fammartino et al., 2007), and *A. thaliana* (Vellosillo et al., 2007). More recently, the expression of genes encoding 9S-LOX was found upregulated following *B. cinerea* infection in grapevine (Gruau et al., 2015), and in *A. thaliana* (Windram et al., 2012), contrasting with the results obtained for *LOX5* in cannabis. Since lipid oxidation mediated by 9S-LOX has been demonstrated to damage plant cell membrane and prompt cell death (Rustérucci et al., 1999), which protects against biotrophic pathogens but promotes necrotrophic pathogens (Govrin and Levine, 2000), we freely postulate that an activation of 9S-LOX might be part of *B. cinerea* attack strategy and that the slight downregulation of *LOX5* observed in cannabis may actually confer an advantage, yet insufficient, against the disease.

### SA-mediated Putative Defenses

Defenses mediated by the SA-pathway are usually effective against biotrophic pathogens and might in contrast strengthen the growth of necrotrophic pathogens that benefit from plant cell death (Thomma et al., 2001). However, the exact role of SA signaling in resistance against the necrotrophic pathogen *B. cinerea* is still unclear and may depend on the plant species (AbuQamar et al., 2017). Therefore, four putative SA-mediated markers were included in this study, encoding cannabis homologous counterparts to three pathogenesis-related (PR) proteins (PR1, PR2, PR5) and one transcriptional co-activator (NPR1).

*PR* genes have been described in many plant families and encode a large variety of pathogen-induced proteins, often with antimicrobial properties. They are usually classified in seventeen groups based on their biochemical and/or biological properties (van Loon et al., 2006). The most abundant PR protein, PR1, is commonly associated with antifungal resistance even though its mode of action is still undetermined. The expression of *PR1* genes often serves as an indicator of plant defense activation (Buchel and Linthorst, 1999). Also commonly used, PR2 proteins are β-1,3-glucanases with several possible structural isoforms and variable hydrolytic activity against β-glucans, which are main components of fungal cell walls. In tomato and tobacco, only class I vacuolar isoforms, presumably like the one in this study, can effectively inhibit fungal pathogens growth *in vitro*. These class I PR2 enzymes act directly by degrading fungal cell wall components, and indirectly by eliciting plant defenses through the release of materials derived from fungal cell walls (Leubner-Metzger and Meins, 1999). Finally, PR5 are cysteine-rich proteins called thaumatins that exhibit broad antifungal effects thanks to a selective membrane-permeabilizing mode of action (Velazhahan et al., 1999).

Our results demonstrated a strong expression upregulation in cannabis infected local leaves for *PR1* and *PR2*, but not for *PR5*. *PR1* expression upregulation following *B. cinerea* infection was notably the strongest of all markers under study, and is also observed in *A. thaliana* (Govrin and Levine, 2002; Ferrari et al., 2003; Nie et al., 2017), lettuce (De Cremer et al., 2013), pepper (Jiang et al., 2018), and tobacco (Frías et al., 2013), but not in grapevine (Gruau et al., 2015). Since *PR1* genes are found in every plant species investigated so far, it is assumed that their function is important enough to be strongly conserved during evolution (van Loon et al., 2006). Our findings bring evidence that *PR1* likely plays indeed an important role in the cannabis-*Botrytis* interaction. Similarly, the upregulation of *PR2* expression in cannabis infected local leaves corroborates results observed during infection of *B. cinerea* in *A. thaliana* (Nie et al., 2017) and grapevine (Gruau et al., 2015), while lack thereof for *PR5* is unexpected (Govrin and Levine, 2002; El Oirdi et al., 2010; Gruau et al., 2015; Nie et al., 2017). Since thaumatins can have a plethora of functions (Velazhahan et al., 1999), it is possible that the cannabis *PR5* reported here is simply not involved in defenses against pathogens.

Finally, the last putative SA-mediated marker in this study was the *NPR1* gene, predicted to encode the regulatory protein Non-expressor of pathogenesis-related genes 1, and its expression was not upregulated in cannabis infected local leaves. In *A. thaliana*, NPR1 acts as a transcription co-activator of a large set of *PR* genes and a key regulator of the plant defense signaling network. In absence of stress, NPR1 oligomers are sequestered in the cytoplasm. Upon pathogen challenge, SA-induced conformational changes and cellular redox state shift lead to NPR1 disassembly. Monomers of NPR1 are then translocated into the nucleus where they recruit TGA transcription factors that bind to promoters of SA-inducible *PR* genes, resulting in their activation (Backer et al., 2019a). Besides these post-translational mechanisms intensively studied in *A. thaliana*, regulation of NPR1 at the transcriptional level has also been observed in tomato and pepper during infection by *B. cinerea* (El Oirdi et al., 2011; Jiang et al., 2018). Indeed, in tomato, *NPR1* expression is upregulated by *B. cinerea* to manipulate the antagonism between the JA- and SA- pathways and strategically promote disease (El Oirdi et al., 2011). As such, and similarly to *LOX5* above, we speculate that the slight downregulation of *NPR1* in cannabis might be an attempt, yet unsuccessful, to counter gray mold development.

### Lack of Systemic Elicitation of Putative Cannabis Defense Genes Expression

The immunity acquired by some plants against reinfection after an earlier pathogen attack has now been studied for almost a hundred years (Chester, 1933). About six decades later, it was demonstrated that beneficial rhizobacteria can also stimulate plant immunity against pathogens (Van Peer et al., 1991). However, the elicitation of such systemic defenses has never been studied in *C. sativa.* Therefore, using the newly developed defense markers presented above, we investigated the potential SAR and ISR elicitation in cannabis, triggered respectively by *B. cinerea* (Figure 4, Supplementary Table S1) and the beneficial rhizobacteria (Supplementary Figure S4). As a lack of substantial elicitation was observed in both cases, the following sections mainly discuss likely reasons and future work directions.

### Lack of SAR-mediated Elicitation of Defense Genes Expression by *B. cinerea*

SAR is defined as an enhanced defensive capacity of the entire plant against a broad range of pathogens that occurs following an earlier localized exposure to a pathogen (Pieterse et al., 2014). This resistance response is usually associated with systemic SA accumulation along with direct transcription activation of defense genes in systemic organs. Consequently, genes encoding SA-inducible PR proteins, such as *PR1*, *PR2* and *PR5*, are often used as hallmarks of SAR elicitation (Thomma et al., 2001), with *PR1* ranking among the best characterized markers (Pieterse et al., 2014). Surprisingly, few researches have focused on SAR responses to gray mold. Three previous studies concluded that *B. cinerea* does not elicit SAR in *A. thaliana* (Govrin and Levine, 2002), nor in lettuce (De Cremer et al., 2013), although it does so in tobacco (Frías et al., 2013). Indeed, a lack of systemic induction was observed for *PR1* and a defensin gene in the first host (Govrin and Levine, 2002), and for 24 genes, including *PR1*, *PAL*, *LOX*, and *ERF1*, in the second host (De Cremer et al., 2013), while systemic induction ranged from about 10-fold to above 100-fold, respectively for *PR5* and *PR1*, in the last host (Frías et al., 2013). Systemic induction of *PR5*, as well as a gene encoding a glutathione transferase, was also shown in *A. thaliana* (Govrin and Levine, 2002), but did not provide protection against subsequent infections, like it did in tobacco (Frías et al., 2013). The time frame for observations in these studies was set from 2 dpi to 8 dpi, and highlighted the importance of allowing sufficient time for SAR initiation in distant organs (De Cremer et al., 2013) and to take into account temporal variations in disease progression (Frías et al., 2013). In cannabis, none of the putative defense genes under study showed a significant expression upregulation in systemic leaves, although the markers did indeed include homologous counterparts to the three SAR hallmark genes presented above and samples were collected at likely appropriate times. On the contrary, downregulations of *LOX5*, *ERF1*, *HEL*, *PR5*, and *NPR1* were observed. Even though it cannot be excluded that these observations are biologically relevant, variations of such small amplitude are unlikely to constitute a substantial SAR-mediated response, especially with the definitive absence of upregulation of *ERF1*, *HEL*, *PAL*, *PR1* and *PR2* that are otherwise strongly activated locally. However, while SAR-mediated responses to gray mold could not be demonstrated in cannabis under the tested conditions, it does not mean that the plant is definitely unable to elicit SAR since the outcome of a secondary infection was not evaluated. Indeed, a subsequent conidia inoculation on the systemic leaves could have potentially resulted in fewer symptoms than the first inoculation, and/or have led to greater gene expression variations that remained undetectable without a second pathogen challenge (priming). Moreover, as only transcriptional responses were experimentally assessed, future work should investigate the potential accumulation of phytohormones that was not explored here. Alternatively, we can also speculate that some plant species and/or cultivars differ in their capacity to activate SAR against *B. cinerea*, or that some fungal isolates differ in their ability to suppress SAR. Indeed, not all cannabis cultivars might be able to activate SAR, in the same way that resistance to gray mold can be cultivar-specific in tobacco (El Oirdi et al., 2010). Inversely, the intraspecific diversity of pathogens also matters when interacting with plants, even for necrotrophic pathogens with a broad host range (Rowe and Kliebenstein, 2010). Since the fungal isolate used in this study was obtained from cannabis plants, it might be interesting to test if isolates from other plants would elicit SAR in cannabis. A similar hypothesis was confirmed in rose (Pie and Brouwer, 1993) and grapevine (Derckel et al., 1999) where *B. cinerea* isolates were more virulent on the plant species that they originated from than isolates obtained from other plant species.

### Lack of ISR-mediated Elicitation of Defense Genes Expression by the Bacteria and Lack of Significant Biocontrol Protection

The gray mold disease caused by *B. cinerea* can negatively impact the yield and quality of cannabis crops and is therefore of major concern for this emerging industry in Canada and in other countries. A few commercial biopesticides, based on beneficial fungi such as *Gliocladium catenulatum* and *Trichoderma harzianum*, are registered in Canada against gray mold in cannabis (Punja, 2018). However, their persistence on above-ground parts of the plant could potentially increase microbial contaminants (Punja, 2018) or mycotoxins (Vujanovic et al., 2020) in the final product. To circumvent this issue, biocontrol agents could be dispensed in the growing medium rather than by foliar sprays, but this option relies on the identification of microorganisms that can elicit systemic defenses in the whole plant (Backer et al., 2019b; Lyu et al., 2019). Consequently, beneficial rhizobacteria, such as widely prevalent *Bacillus* and *Pseudomonas* strains, are expected to potentially offer the combined benefits of ISR-mediated disease control, plant growth promotion, and cannabinoid yield enhancement in cannabis production (Lyu et al., 2019). In this study, we examined the application of two plant growth promoting *Bacillus* strains and two *Pseudomonas* strains as root treatments to control gray mold and to elicit ISR in cannabis plants. Our results suggested that the biocontrol protection was rather inefficient in the conditions tested. None of the bacteria-treated plants ultimately differed from the water-treated plants as all infected leaves were necrotic after a week, regardless of the treatment (Figure 1). This conclusion was also supported by the lack of putative defense genes expression induction or priming by the rhizobacteria (Supplementary Figure S4), even if some promoted plant growth (Supplementary Figure S1). These findings are not entirely unexpected since the ability of a plant to develop ISR in response to certain rhizobacteria depends on the specificity of their mutual interactions (Beneduzi et al., 2012), even though bacteria isolated from one plant species can notoriously promote growth and induce systemic resistance in other crop species (Backer et al., 2019b). For instance, *P. simiae* WCS417r was isolated from the wheat rhizosphere and elicits ISR in *A. thaliana*, grapevine, radish, banana, bean, carnation, and tomato, but not in eucalyptus or tobacco (Berendsen et al., 2015). The same bacterium proved here unable to reduce gray mold symptoms (Figure 1) or to elicit ISR-enhanced genes expression in cannabis (Supplementary Figure S4). The other *Pseudomonas* strain tested here was *P. synxantha* LBUM223, a phenazine antibiotic producer (Arseneault et al., 2014). Antibiotics from *Pseudomonas* spp. have been reported to trigger ISR in *A. thaliana* against *Hyaloperonospora arabidopsidis* (formerly *Peronospora parasitica*) (Iavicoli et al., 2003) and in tomato against *B. cinerea* (Audenaert et al., 2002), but such effects were not observed in cannabis. Finally, the two *Bacillus* strains under study were *B. velezensis* LBUM279 and *B. subtilis* LBUM979, which produce several antibiotics and promote cannabis growth (Supplementary Figure S1). These bacteria were chosen since ISR-eliciting *Bacillus* strains typically also promote plant growth (Kloepper et al., 2004). However, this association of traits could not be corroborated here.

As this is the first documented attempt to control any disease on cannabis plants by ISR with beneficial rhizobacteria, many avenues remain yet to be explored. Besides relying on plant-specific interactions, the efficiency of biocontrol agents against gray mold is known to be influenced by several other factors, including environmental conditions, inoculum stability, concentration and quality, timing of application, and susceptibility of *B. cinerea* isolates (Nicot et al., 2016). Here, the rhizobacteria were inoculated one week before pathogen challenge on the basis that ISR is typically triggered in *A. thaliana* during the first 7 days and lasts at least 21 days following root treatment by *P. simiae* WCS417r (Van Wees et al., 1999). However, considering how *B. cinerea* is difficult to control, any single management measure is unlikely to succeed (Williamson et al., 2007). In an attempt to improve the efficiency of the bacterial treatments used in this study, combined applications of each *Bacillus* sp. with each *Pseudomonas* sp. were also tried, since bacterial consortia can potentially benefit from complementary ecological requirements and modes of action (Sarma et al., 2015). Unfortunately, none of the four consortium treatments differed significantly from the single-bacteria treatments, suggesting a lack of synergism under the tested conditions. So far, the most investigated consortium of *Pseudomonas* spp. is with *Bacillus* spp. and many studies conducted on these combinations have reported a synergistic biocontrol effect (Hol et al., 2013). However, conflicting results have also been reported, supporting either positive or negative interactions, for example regarding biofilm formation (Powers et al., 2015; Ansari and Ahmad, 2019). Altogether, these results confirm that the outcome of combinations of *Pseudomonas* spp. with *Bacillus* spp. likely depends on their compatibility at the species and/or strain level. Our work highlights the importance of gaining a better understanding of complex interactions in integrated pathological systems to be able to identify robust biocontrol agents for new crops.

## Conclusion and Perspectives

To conclude, our results demonstrate that five out of eight putative defense genes, namely *ERF1*, *HEL*, *PAL*, *PR1*, and *PR2*, are strongly upregulated locally in cannabis leaves infected by gray mold. These results validate that our model is useful to detect cannabis responses to a pathogen attack at the molecular level, a research area yet to be explored. Therefore, we propose that *ERF1*, *HEL*, *PAL*, *PR1*, and *PR2* can be used as reliable markers of cannabis inducible defenses and/or stress responses pathways. Further functional studies with these markers could pave the way to rapid assessment of suitable control methods and improvement of assisted cultivars breeding.

## Data Availability Statement

The raw data supporting the conclusions of this article will be made available by the authors, without undue reservation, to any qualified researcher.

## Author Contributions

CB, AN, DJ, and MF contributed to conception and design of the study. CB, GC, and AN performed the experiments. CB analyzed the data and drafted the manuscript. DJ and MF contributed reagents, equipment, and funds. All authors revised the manuscript, read, and approved the submitted version.

## Conflict of Interest

The authors declare that the research was conducted in the absence of any commercial or financial relationships that could be construed as a potential conflict of interest.
