# Supplementary material for: Expression of Putative Defense Responses in Cannabis Primed by Pseudomonas and/or Bacillus Strains and Infected by Botrytis cinerea
Source: Front Plant Sci. 2020 Nov 25;11:572112. doi: 10.3389/fpls.2020.572112 (PMC7723895; doi:10.3389/fpls.2020.572112)
Supplement: Supplementary file 1 [file Data_Sheet_1.PDF]

Supplementary Table S1. Effects of harvest time and *B. cinerea* treatment on the expression of putative defense genes in cannabis leaves. (A) Changes over time of genes expression in uninfected, systemic, and local leaves. Fold changes are scaled relatively to the uninfected leaves at 2 dpi (expression level of 1.0). Significant differences from 2 dpi are in bold for each type of leaves separately (Wald chi-square test with Benjamini-Hochberg correction, α=.05). *P* values are replaced by NS when post-hoc comparisons were not carried out since the time had no significant effect for the type of leaves considered (*LOX5* uninfected leaves *P*=.49, systemic leaves *P*=.08, local leaves *P*=.49; *PR1* uninfected leaves *P*=.72, systemic leaves *P*=.30, local leaves *P*=.72; *PR2* uninfected leaves *P*=.07, systemic leaves *P*=.85; *PR5* uninfected leaves *P*=.49; *NPR1* uninfected leaves *P*=.17). **(B)** Effect of *B. cinerea* treatment on genes expression at each harvest time. Fold changes are scaled relatively to the uninfected leaves at each harvest time (expression level of 1.0). Significant differences from uninfected leaves are in bold for each time separately (Wald chi-square test with Benjamini-Hochberg correction, α=.05). Upregulation rises above 1.0 and downregulation falls below. Means of 36 independent biological replicates with 3 technical replicates.

A. Effect of harvest time on genes expression

	Uninfected leaves			Systemic leaves			Local leaves		
**Gene**	**2 dpi**	**4 dpi**	**7 dpi**	**2 dpi**	**4 dpi**	**7 dpi**	**2 dpi**	**4 dpi**	**7 dpi**
LOX5	1.0	1.2	1.1	0.6	0.8	0.9	0.9	0.8	0.8
	P value	NS	NS	*P* value	NS	NS	*P* value	NS	NS
ERF1	1.0	**4.9**	**6.6**	0.9	**2.6**	**5.0**	17.1	**45.9**	**59.6**
	P value	**<.001**	**<.001**	*P* value	**<.001**	**<.001**	*P* value	**<.001**	**<.001**
HEL	1.0	**1.8**	**2.3**	0.6	**1.0**	**1.9**	11.2	**37.1**	**96.0**
	P value	**.03**	**.002**	*P* value	**.03**	**<.001**	*P* value	**<.001**	**<.001**
PAL	1.0	1.7	**4.2**	1.5	1.3	**4.2**	25.3	47.1	**125.6**
	P value	.17	**<.001**	*P* value	.92	**.007**	*P* value	.06	**<.001**
PR1	1.0	1.6	1.9	1.4	1.1	4.5	373.4	638.3	536.3
	P value	NS	NS	*P* value	NS	NS	*P* value	NS	NS
PR2	1.0	1.0	0.5	0.7	0.6	0.7	4.6	**15.4**	6.2
	P value	NS	NS	*P* value	NS	NS	*P* value	**.001**	.98
PR5	1.0	1.1	0.9	0.7	0.9	0.5	0.8	0.6	**0.1**
	P value	NS	NS	*P* value	.12	.07	*P* value	.19	**<.001**
NPR1	1.0	1.0	0.8	0.6	**0.8**	**0.5**	0.9	0.9	**0.5**
	P value	NS	NS	*P* value	**.01**	**.02**	*P* value	.79	**<.001**

B. Effect of *B. cinerea* treatment on genes expression

	2 dpi			4 dpi			7 dpi		
**Gene**	**Uninfected**	**Systemic**	**Local**	**Uninfected**	**Systemic**	**Local**	**Uninfected**	**Systemic**	**Local**
LOX5	1.0	**0.6**	0.9	1.0	**0.7**	**0.7**	1.0	0.8	**0.7**
	P value	**.001**	.36	*P* value	**.001**	**.001**	*P* value	.28	**.02**
ERF1	1.0	0.9	**17.1**	1.0	**0.5**	**9.3**	1.0	0.7	**9.0**
	P value	.80	**<.001**	*P* value	**.02**	**<.001**	*P* value	.30	**<.001**
HEL	1.0	**0.6**	**11.2**	1.0	**0.6**	**20.5**	1.0	0.8	**41.1**
	P value	**.04**	**<.001**	*P* value	**.04**	**<.001**	*P* value	.40	**<.001**
PAL	1.0	1.5	**25.3**	1.0	0.8	**28.1**	1.0	1.0	**30.2**
	P value	.69	**<.001**	*P* value	.69	**<.001**	*P* value	.69	**<.001**
PR1	1.0	1.4	**373.4**	1.0	0.7	**394.2**	1.0	2.4	**286.7**
	P value	.80	**<.001**	*P* value	.80	**<.001**	*P* value	.35	**<.001**
PR2	1.0	0.7	**4.6**	1.0	0.6	**15.5**	1.0	1.4	**12.0**
	P value	.25	**<.001**	*P* value	.11	**<.001**	*P* value	.70	**<.001**
PR5	1.0	**0.7**	0.8	1.0	0.8	**0.6**	1.0	**0.6**	**0.1**
	P value	**.01**	.12	*P* value	.14	**<.001**	*P* value	**<.001**	**<.001**
NPR1	1.0	**0.6**	0.9	1.0	**0.7**	0.9	1.0	**0.6**	**0.5**
	P value	**<.001**	.23	*P* value	**.01**	.23	*P* value	**<.001**	**<.001**
