# Supplementary figures and images for: Expression of Putative Defense Responses in Cannabis Primed by Pseudomonas and/or Bacillus Strains and Infected by Botrytis cinerea
Source: Front Plant Sci. 2020 Nov 25;11:572112. doi: 10.3389/fpls.2020.572112 (PMC7723895; doi:10.3389/fpls.2020.572112)

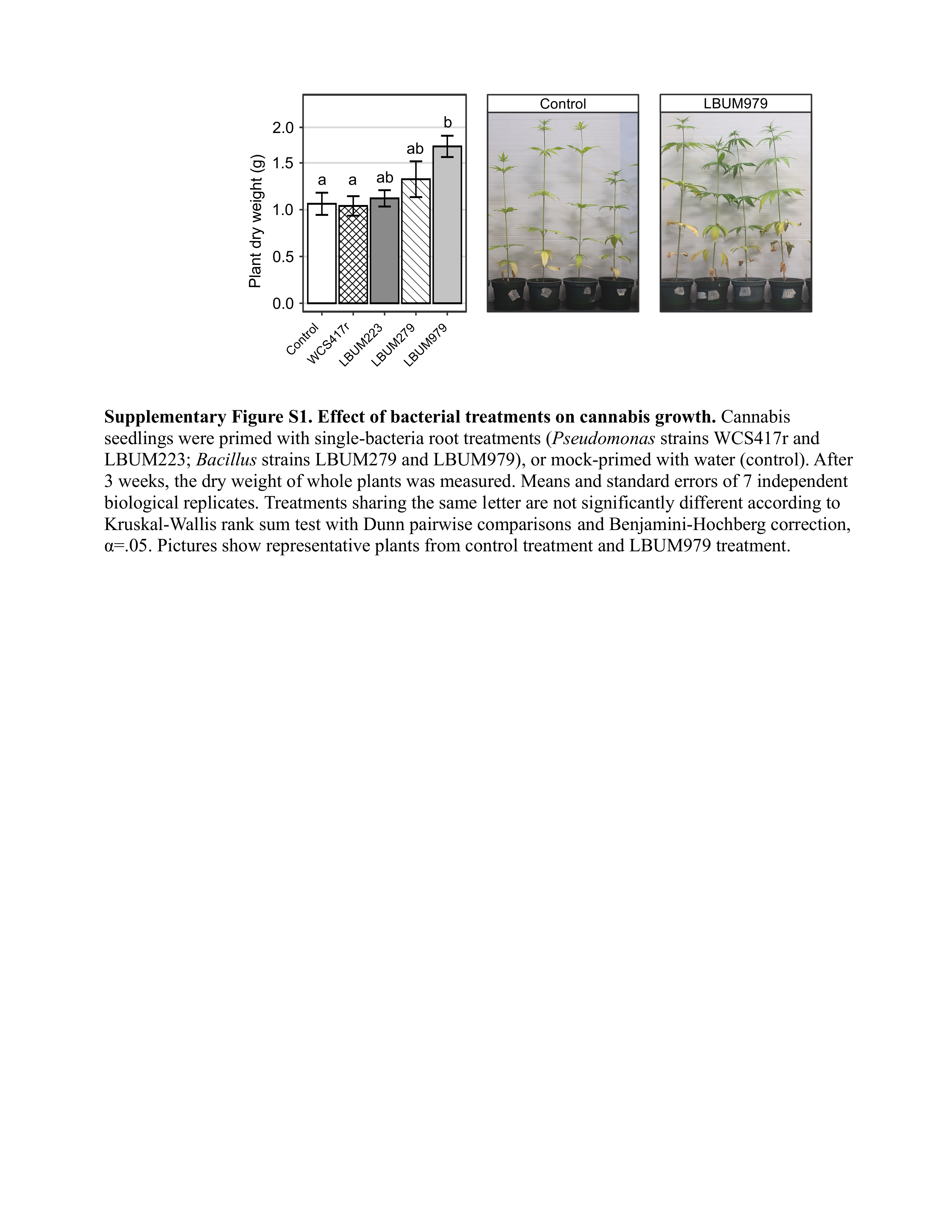

Supplement: Supplementary file 2 [file Image_1.TIFF]

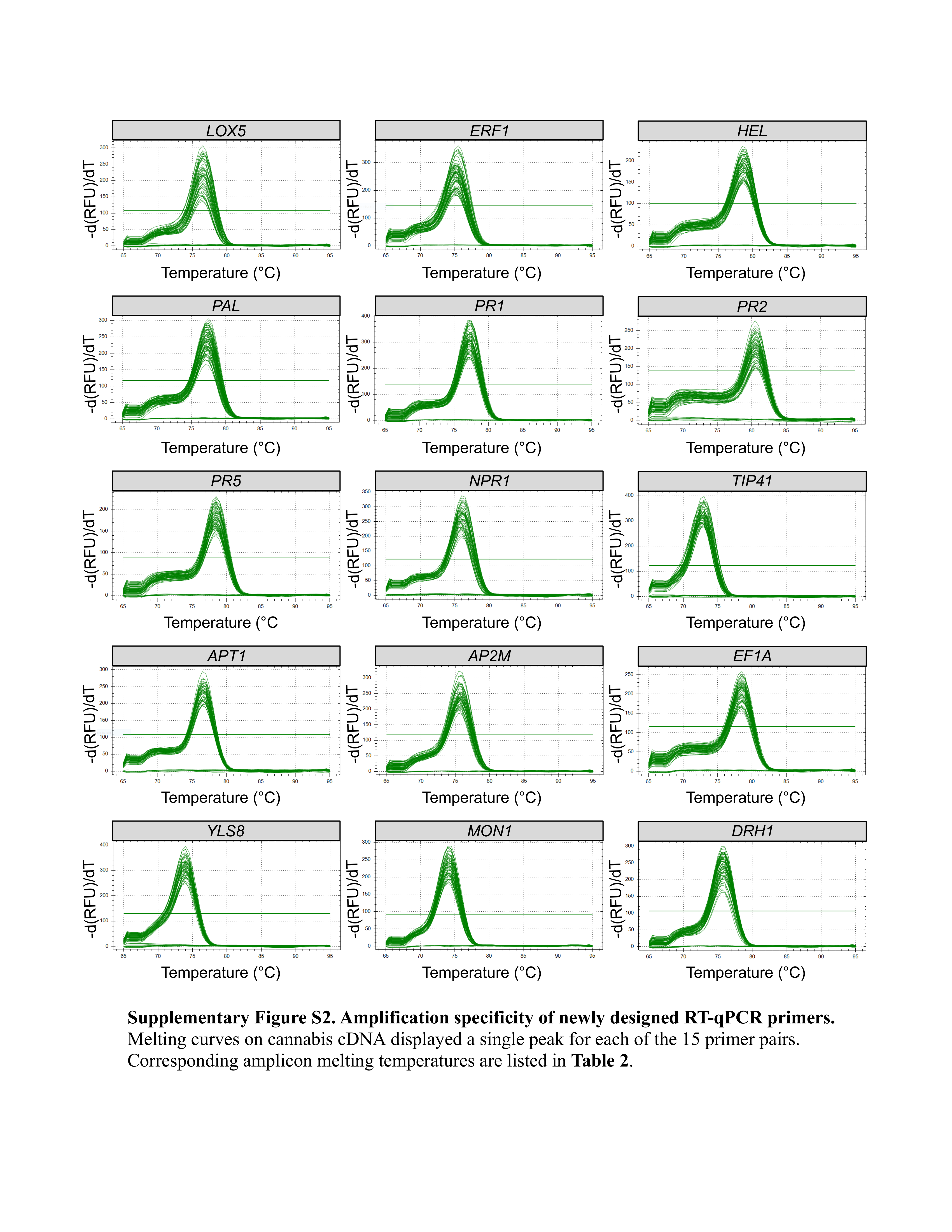

Supplement: Supplementary file 3 [file Image_2.TIFF]

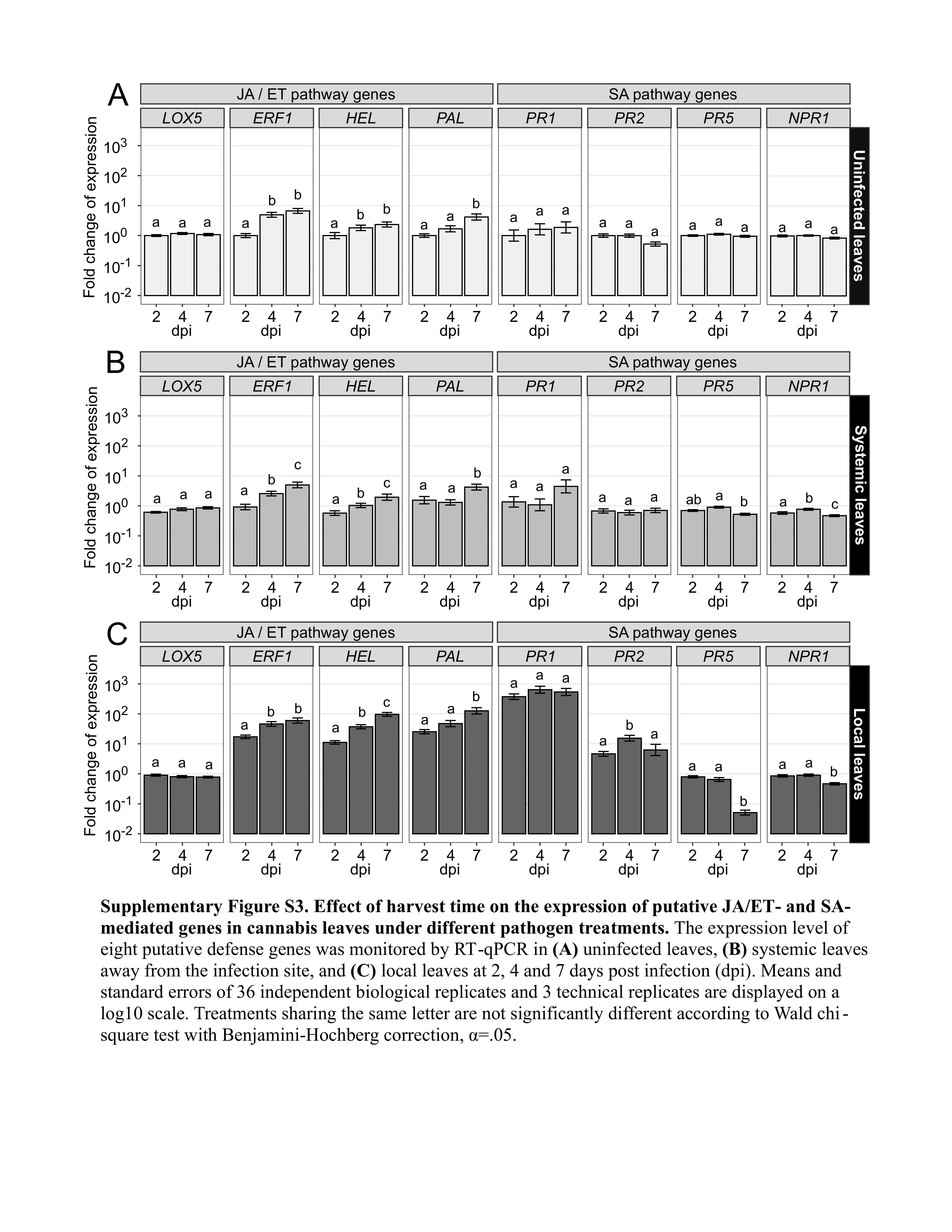

Supplement: Supplementary file 4 [file Image_3.TIFF]

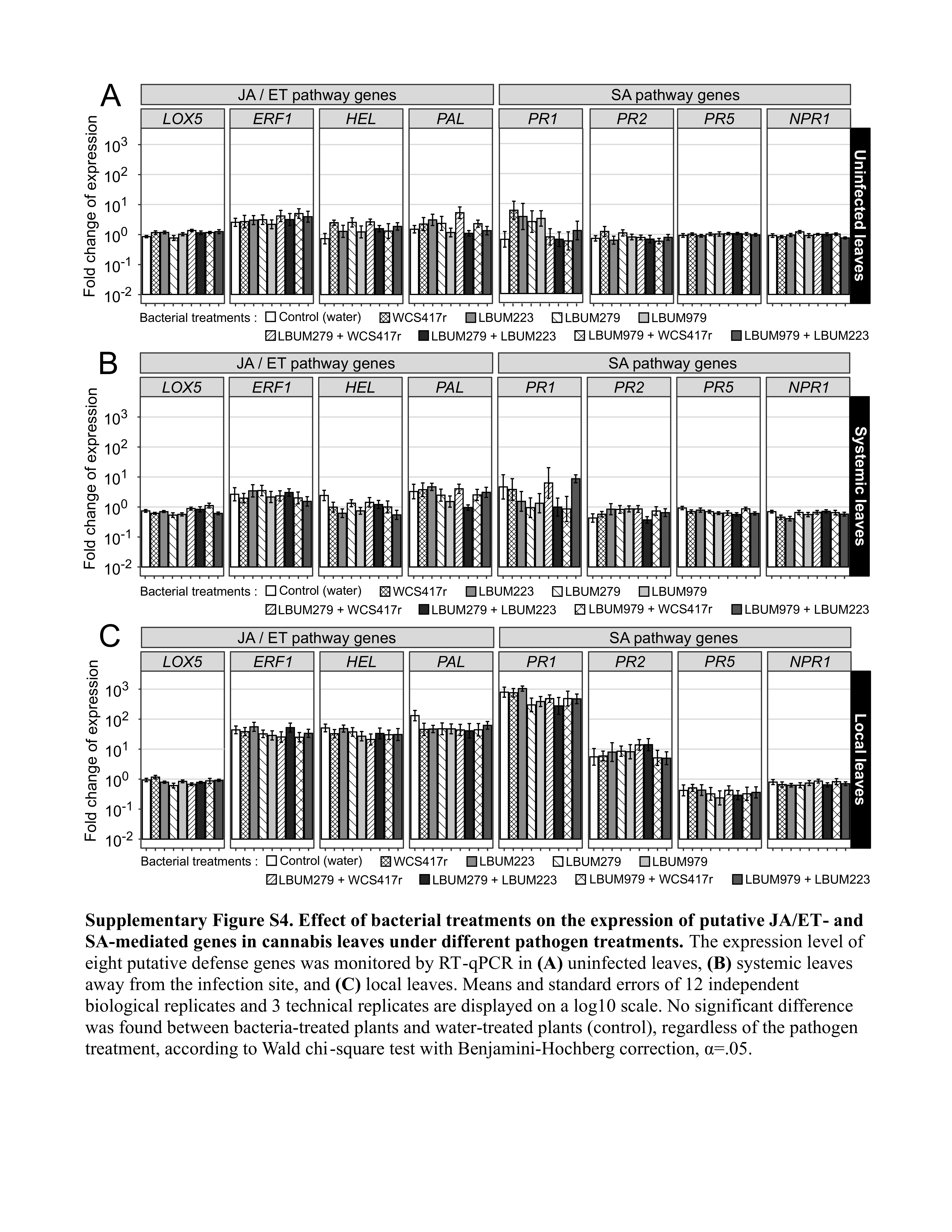

Supplement: Supplementary file 5 [file Image_4.TIFF]
